# PowderBot: An automated device for decision-making in crop breeding programs based on DNA extraction from seed powder

**DOI:** 10.1016/j.ohx.2025.e00706

**Published:** 2025-09-22

**Authors:** H. Díaz, E. Macea, R. Escobar, S. Beebe, J. Tohme, B. Raatz

**Affiliations:** The Alliance of Bioversity International and CIAT, Km 17 Recta Cali-Palmira, Apartado Aereo 7613, Cali, 763537, Colombia

**Keywords:** Mechatronics, Plant breeding, DNA analysis, Genotyping

## Abstract

This paper presents the design, construction, operation, and evaluation of PowderBot, a purpose-built, open-source, low-cost machine (∼US$ 5000) that automates DNA extraction from ungerminated seeds. The device drills into the seed cotyledon, where the genetic information of the prospective plant is stored. It then transfers the pure, powdered samples directly to well-plates for analysis. This reduces time and other research resources and can accelerate crop varietal improvement, ultimately contributing to more efficient and successful crop breeding programs. At CIAT́s campus, we have validated the method for obtaining seed-tissue material from common beans for DNA extraction and subsequently genotyping agronomically interesting lines, using the bc-3 molecular marker. Three genotyping trials were carried out using this method, which generated consistent results, regardless of the number of perforations made to the seed. This leads us to infer that the method works effectively and can be applied for marker assisted selection (MAS) in bean and other crop breeding programs. Finally, germination and vigor tests indicated the sampling process did not significantly compromise perforated seed germination rate, physiological quality or viability.

## Graphical abstract

**Figure:**
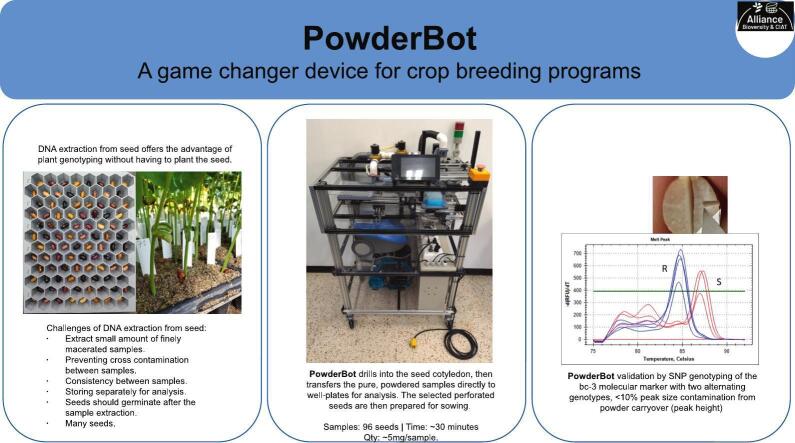

## 1 Hardware in context

Plant breeding aims to develop new crop varieties with desirable traits, such as high yield, abiotic stress tolerance, disease resistance, easier processing, and improved nutritional content [1]. Modern plant breeding techniques utilize genetic analysis (DNA analysis), to inform marker-assisted selection (MAS), genomic selection, and gene editing. DNA extraction is the initial step for DNA-based molecular marker analysis, offering the advantage of selecting new crop varieties more efficiently replacing some phenotypic analyses. This requires rapid extraction of DNA from large numbers of samples involving many crop plant tissue types. DNA is usually extracted from lyophilized leaf samples. DNA extraction from seed offers the advantage of plant genotyping without having to plant the seed. Seed will still germinate after the analysis, enabling selection of seed before planting, thereby increasing resource efficiency.

**Fig. 1:**
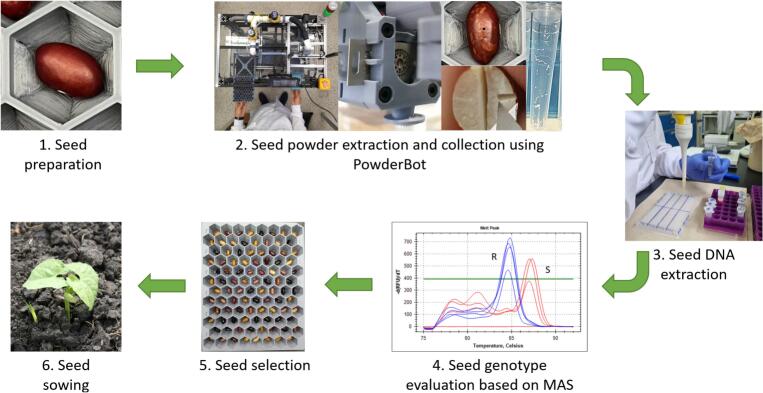
Workflow for which the experimental hardware (PowderBot) was developed.

In our case, we aim to extract DNA from common bean (Phaseolus vulgaris) seeds, which are quite large and hard. Seed DNA extraction requires drilling into the cotyledon, where the genetic information of the prospective plant is stored. Drilling is performed to obtain 3 to 10 mg of seed powder, which is then deposited in 96-well plates followed by a standardized DNA extraction protocol. The main challenge lies in extracting small amounts of finely macerated samples from many seeds, while ensuring consistency between samples, preventing contamination from seed skin material or contamination of seed powder between samples, and storing them separately in plates. The selected perforated seeds are then prepared for sowing Fig. 1.

There is a plethora of DNA extraction protocols available. Seed DNA extraction protocols are available for many crops and different downstream applications, e.g. [2,3]. Drilling protocols have been published in soy bean [4] or barley [5].

Automated genetic analysis of seed has been exclusively developed and used commercially by major seed companies. Methods include seed chipping, laser cutting, grinding, leaching, and others, depending on the evaluated seed species. Private companies like Bayer use various techniques to reduce seed preparation and study times, in crops like corn and soybean, for subsequent laboratory evaluation. These techniques allow them to focus on field trials with selected material, making them an invaluable resource for these companies. However, these instruments are not commercially available. The cost of these techniques and the product protection patents associated with them, e.g. [6], make them unavailable to molecular breeding research groups. Unfortunately, the exact cost of these techniques and the level of patent protection are not disclosed, making it difficult to determine their accessibility to other research groups.

Therefore, we present the design, construction, operation, and evaluation of PowderBot, a purpose-built, open-source, low-cost machine (*∼*US$ 5000) that automates the extraction of DNA from ungerminated seeds. The device can be also used for other crop seeds of various shapes and sizes, such as maize, wheat, and rice. Extracting DNA from ungerminated seeds for marker-assisted selection prior to planting can save a significant amount of time and resources on a large scale, selecting the best germplasm. The system has also been tested with other seeds including maize, rice, and wheat, with successful preliminary results (Table 1).

**Table 1:** Specifications table.

Hardware name	*PowderBot*
Subject area	• Engineering • Biological sciences • Environmental, planetary, and agricultural sciences
Hardware type	• Mechatronics • Biological sample handling and preparation
Open-source license	CC BY-NC-ND 4.0
Cost of hardware	*∼ US$ 5,000.00*
Source file repository	https://doi.org/10.17632/czf4t7mfdr.2

## 2 Hardware description

**Fig. 2:**
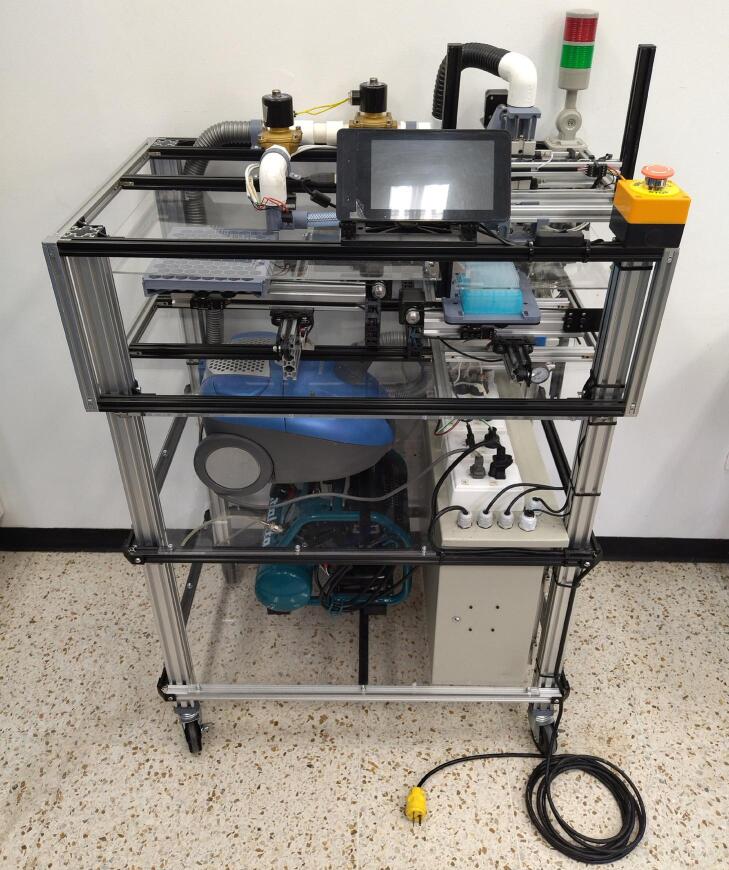
PowderBot fully assembled.

The PowderBot is an automated machine for drilling crop seed samples to extract seed powder for DNA extraction that enables researchers to reduce time and resources and accelerates the breeding process. A complete assembly of the machine is shown in Fig. 2.

The functional and design-related specifications of the PowderBot are listed below.

1. Seed Size:
  - 5–20 mm in diameter.
2. Throughput Capacity:
  - Up to 96 samples per run.
  - Modular trays from 1 to 96-sample batch processing.
3. Extraction Time:
  - 30–60 min per run depending on seed perforations.
4. Seed Powder Extraction:
  - 5–20 mg depending on seed perforations.
5. Automation Level:
  - Semi-automated: Manual plates positioning, automatic powder extraction.
6. Interface & Software:
  - 7-inch touchscreen interface.
  - Web UI app, custom positioning, 1 to 96-sample extraction.
7. Footprint & Power:
  - Dimensions: 1200 × 800 × 600 mm (HxLxW).
  - Power: 100–127 V AC, 50/60 Hz.

PowderBot is composed of 6 modular sub-systems.

1. The first subsystem is called *Pressure* and is responsible for maintaining the required air flow on the entire system.
2. The second subsystem is called *Suction* and overseas provides negative pressure or vacuum to move the seeds from seed plate to seed sampling capsule and vice versa.
3. The third subsystem is named *Seed* and is a belt-driven motion platform that controls the XY-position of the seed plate.
4. The fourth subsystem is called *Powder* and handles the plate in which the seed powder is collected. It is built in the same way as the *Seed* subsystem.
5. The fifth subsystem is called *Sampling* and is responsible for extracting the sample from the seeds in powder form.
6. The sixth subsystem is the *Control Box*, which contains all the electronics and circuitry components.

A detailed description of each subsystem follows. (Please refer to the Designator column inside the Bill of Materials [7] document for a better understanding of the list of materials, [letter number]).

### 2.1 Pressure subsystem

**Fig. 3:**
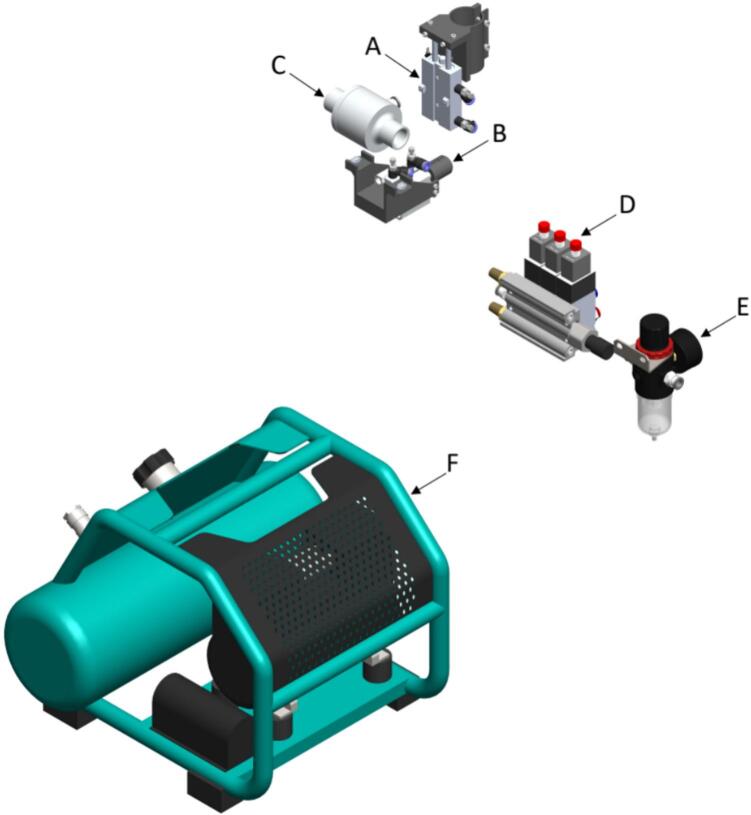
Pressure subsystem (A) Pneumatic Cylinder − 50 mm Stroke, (B) Pneumatic Cylinder − 20 mm Stroke, (C) 1-inch Air Operated Conveyor, (D) Pneumatic Solenoid Valve − Triple Set, (E) Air Filter Pressure Regulator, (F) Air Compressor − 0.7 CFM.

The pressure subsystem is composed of an air compressor [A30] with the power of 350 W, 07 SCFM @ 90PSIG, with a tank size of 1 Gal (3.8L). This provides a pressure load through flex tubes [A52] to move three pneumatic cylinders [A65, A66, A2]. These are controlled by electro-valves each powered by a 110 VAC (Volts Alternating Current) solenoid [A67]. The first pneumatic cylinder [A66] is a dual-acting cylinder with a 16 mm bore, a 50 mm stroke length, and aluminum dual rod, whose function is to position the seed-sampling injector [D30] with the seed-sampling capsule [D28]. The second pneumatic cylinder [A66] is also a dual-acting cylinder with a 20 mm single-rod stroke. Its function is to move the actuator that fixes the seed inside the seed sampling capsule [D28]. The third cylinder is an air operated conveyor [A2] made in aluminum that is used to boost the seed movement from the seed plate [D20] to the seed sampling capsule [D28]. An air filter pressure regulator [A31] is also needed to regulate the air pressure to all the air flow systems. An image of the Pressure subsystem is shown in Fig. 3.

### 2.2 Suction subsystem

**Fig. 4:**
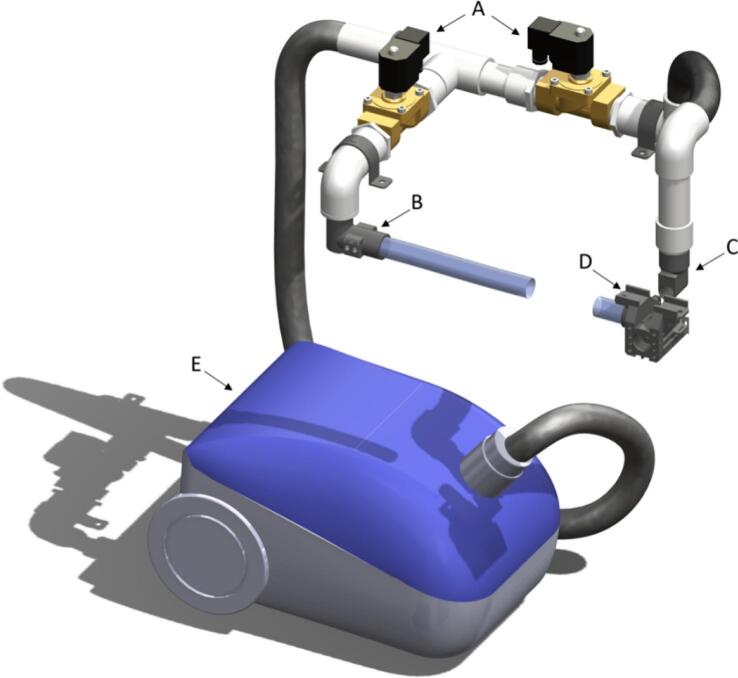
Suction subsystem (A) 1-inch Solenoid Valve − 110 AC, (B) Seed Plate Injector, (C) Seed Sampling Injector, (D) Seed Sampling Capsule, (E) Air Vacuum − 1400 W.

The suction subsystem is based on a 1400 W vacuum cleaner [A34] that generates negative air pressure or vacuum airflow of 50–100 CFM (Cubic Feet per Meter) (depending on the set level, which must be the maximum). This vacuum cleaner is connected to a 1-inch PVC pipe [A7] of 1-meter through a flex tube [A52], 1 T element [A10], 3 elbows [A5], and 1 reduction junction from 1 to inch to ½-inch [A9]. After that, there are 2 brass electric solenoid valves [A11] controlled by 110 VAC, which limits air flow in the direction needed, and 4-male connectors [A6], two for each electro valve. The seeds are moved by the suction subsystem from the seed plate injector [D21], which is equipped with two infrared sensors [A57] to detect seed direction and seed passage. The seed then goes to the seed sampling capsule [D28], which has an infrared LED transmitter that detects if the seed is positioned inside it, then sends a signal to an infrared LED receptor located inside the seed sampling injector [D30]. This allows the system to be informed if the seed is in the correct position inside seed-sampling capsule [D28] for sample extraction. An image of the Suction subsystem is shown in Fig. 4.

### 2.3 Seed subsystem

**Fig. 5:**
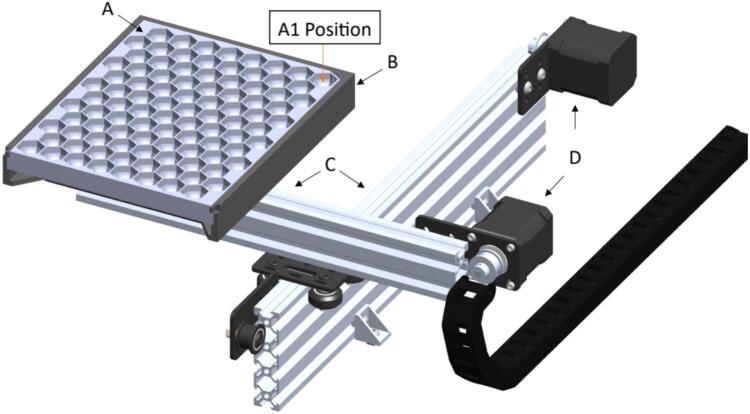
Seed subsystem (A) Seed Plate, (B) Seed Plate Support, (C) 2080 V-Slot Aluminum Profile − 400 mm and 2040 V-Slot Aluminum Profile − 300 mm (D) NEMA 17 Stepper Motors.

The seed subsystem has a seed plate [D20] that stores up to 96 seeds in 8 rows named by sequence of letters from A to H, and 12 columns by the sequence of numbers from 1 to 12, with a honeycomb design for space optimization and is hosted by the seed plate support [D24]. This custom-made part was manufactured using 3D printing and PLA (Polylactic Acid) material. The movement for positioning this plate is made on two sliding aluminum v-slot linear rails, the first one [A26] being 400 mm long and the second [A25] being 300 mm long. Also, there are two stepper motors that allow X and Y perpendicular directions. These stepper motors [A62] are NEMA 17 bipolar 4-lead with 2A rated current/phase, 59ncm (84 oz.in) holding torch, 1.8-degree step angle, and 12–24 V (suggest 24 V) driving voltage. The home position, defined as (0,0), is determined by two limit switches [O22], one for each axis. This position is where the user manually inserts or removes the seed plate. Once the home position is reached, and the user correctly places the seed plate, the system is ready to move this plate to the initial position A1 to start a new sampling process. Every single seed is transported from this plate to the seed sampling capsule [D28] and carried back again to its unique position. An image of the seed subsystem is shown in Fig. 5.

### 2.4 Powder subsystem

**Fig. 6:**
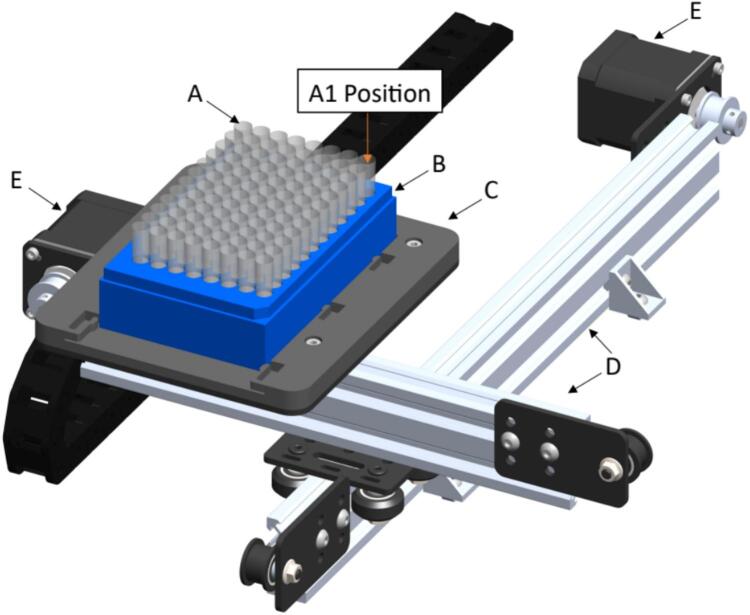
Powder subsystem (A) Test Tubing (B) Test Tubing (Receptacle) (C) Powder Plate Support (D) 2040 V-Slot Aluminum Profile − 400 mm and 2040 V-Slot Aluminum Profile − 250 mm (E) NEMA 17 Stepper Motor.

The powder subsystem handles the plate in which the seed powder is collected. It is built in the same way as the seed subsystem. The differences are in the length of the two-sliding aluminum 2040 (is 20 mm wide and 40 mm high) v-slot (beveled edge) linear rails, one [A24] is 400 mm long and the other [A22] 250 mm. The sample test-tubing receptacles [S1] are standard laboratory 96-tube plates, with 1.1 mL polypropylene natural color tubes in strips of 8, provided by USA Scientific, Inc. with standard measurements, which are used to program the movement in the system using stepper motors. The powder plate support [D18] just gives a guidance for solid positioning for mounting the test tubing receptacle [S1] on the PowderBot. An image of the powder subsystem is shown in Fig. 6.

### 2.5 Sampling subsystem

**Fig. 7:**
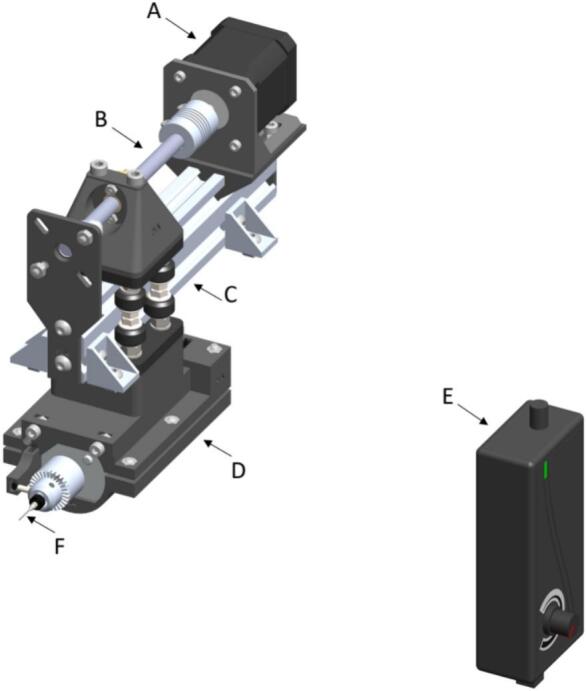
Sampling subsystem (A) NEMA 17 Stepper Motor, (B) Lead Screw Rod with Copper Nut − 100 mm (C) 2040 V-Slot Aluminum Profile − 200 mm (D) Hand Drill Support (E) Adjustable Power Supply − 5 To 12 DC, (F) Drill Bit − 0.7 mm.

The sampling subsystem comprises a linear prismatic joint in the Y-axis that moves an aluminum mini electric portable handheld drill with 5A DC5V-12 V power supply for speed control [A29], which drills a small hole into the seed. The movement for positioning is made on a sliding aluminum 2040 v-slot linear rail, with a length of 200 mm [A21]. A lead screw controls the linear motion for drill positioning to move the drill into the seed using a stepper motor [A62]. The seed is fixed in customized part called seed-sampling capsule, also made with a 3D printer. The seed is held by the pneumatic cylinder [A65] described in the Pressure subsystem (Fig. 2.B). A 0.7 mm bit [A46] is used to perforate the seed by activsting the drill and the stepper motor movement. As the device drills the hole in the seed, the sample powder falls into the sample tube. The seed is released by retracting the pneumatic cylinder [A65] and it is transported from the seed-sampling capsule to the seed plate by activating reverse airflow, which also allows the cleaning of the system including pipes and funnel, while the drill is returned to its initial position determined by a limit switch [O22]. After the cycle has been completed the seed plate and the powder plate are moved to the next position for extracting the next seed sample. An image of subsystem 5 is shown in Fig. 7.

### 2.6 Control subsystem

**Fig. 8:**
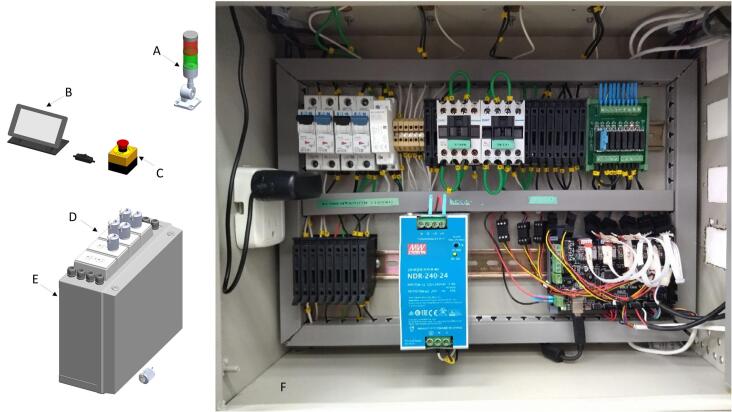
Control subsystem (A) LED Signal Tower − 110 AC, (B) Touchscreen Display – 7-inch', (C) Emergency Stop Button, (D) Outlet and Wall Plate, (E) Polycarbonate Washdown Enclosure, (F) Circuitry.

PowderBot is controlled by two main boards, one of which is the Raspberry Pi [A71], and the other the MKS Gen 1.4 microcontroller [A48]. Further electronic components are for user interface, including the touchscreen [A81, A82], tower light [A61], emergency button [A49], power supply [A69], fuses relay board module [A54, A55], etc. Most of these elements are packed inside a polycarbonate washdown enclosure [M9] and fixed using DIN rails [A44]. In the electrical diagram documentation is a detailed explanation of how the circuitry connection works (Circuit Diagram) [7]. An image of subsystem 6 is shown in Fig. 8.

### 2.7 Software and application

**Fig. 9:**
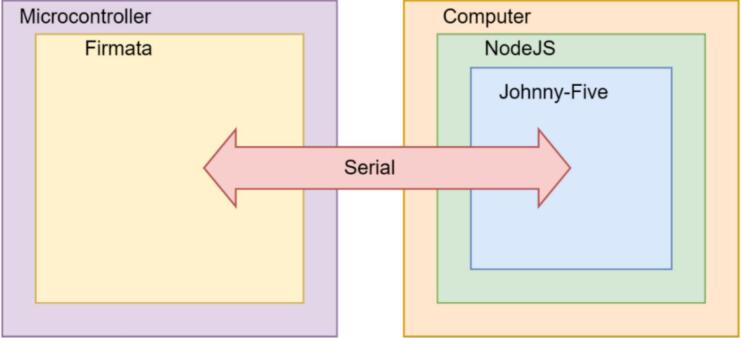
Software protocol implemented for PowderBot web app.

PowderBot is operated by a web application based on JavaScript coding. A Serial port communication is established to send instructions from the microcontroller using Firmata firmware to the Raspberry Pi through Johnny-Five programming framework (Fig. 9). The commands vary from plate positioning, quantity of seeds to be processed and to starting the procedure.

In a research laboratory context, PowderBot can be used to:

• Significantly streamlining the process of DNA sampling from seed tissue.
• Enhance the capacity to process a large number of samples, providing a robust foundation for genetic studies and breeding programs.
• Increase the speed and scale of molecular analysis in a wide range of agricultural research settings.
• Take advantage of its non-destructive process which allows crop breeders to evaluate and select seeds based on its genetic information.
• Improve the capacity building due to the cost-benefit and open source of this development.

## 3 Design files

All relevant design files required to reproduce this work. (Table 2).

**Table 2:** Design files summary.

**Design file name**	**File type**	**Open-source license**	**Location of the file**
3DprintFiles.rar	STL	CC BY NC 3.0	https://doi.org/10.17632/czf4t7mfdr.2
LaserCut.rar	DXF	CC BY NC 3.0	https://doi.org/10.17632/czf4t7mfdr.2
PowderBot1.0.igs	IGES	CC BY NC 3.0	https://doi.org/10.17632/czf4t7mfdr.2
Treehouse.pdf	PDF	CC BY NC 3.0	https://doi.org/10.17632/czf4t7mfdr.2
BillOfMaterials.xlsx	MS Excel file	CC BY NC 3.0	https://doi.org/10.17632/czf4t7mfdr.2
BuildInstructions.pdf	PDF	CC BY NC 3.0	https://doi.org/10.17632/czf4t7mfdr.2
OperationInstructions.pdf	PDF	CC BY NC 3.0	https://doi.org/10.17632/czf4t7mfdr.2
CircuitDiagram.pdf	PDF	CC BY NC 3.0	https://doi.org/10.17632/czf4t7mfdr.2
PowderBot.rar	RAR	CC BY NC 3.0	https://doi.org/10.17632/czf4t7mfdr.2
PowderBot_HD_2.mp4	MP4	CC BY NC 3.0	https://doi.org/10.17632/czf4t7mfdr.2

**3DprintFiles.rar** is a zipped folder containing all the CAD (STL) files for 3D printing the components of PowderBot.

**LaserCut.rar** is a zipped folder containing DXF files for laser cutting acrylic parts.

**PowderBot1.0.IGS** is an IGES file to explore all the PowderBot components in one assembly.

**Treehouse.pdf** is a pdf document for assembly hierarchy.

**BillOfMaterials.xlsx** is a Microsoft Excel document for the detailed bill of materials.

**BuildInstructions.pdf** is a pdf document for standalone use as a construction manual for PowderBot.

**OperationInstructions.pdf** is a pdf document for standalone use as an operation manual for PowderBot.

**CircuitDiagram.pdf** is a pdf document for standalone use as a detailed circuitry connection for PowderBot.

**PowderBot.rar** is a compressed file that includes all the programming code used to operate PowderBot.

**PowderBot HD 2.mp4** is a mp4 video file format that shows the seed sample extraction process made by PowderBot.

## 4 Bill of materials

The detailed bill of materials is included as a supplemental Excel document in the source file repository at https://doi.org/10.17632/czf4t7mfdr.2.

## 5 Build instructions

To build the PowderBot machine, some basic builder and circuitry skills are needed, including basic tools such as a cutter, soldering iron, calipers, inch and metric Allen key sets, pliers, and other small hand tools, and familiarity with 3D printing. In the following build instructions, every printed part and component is given an identifier that refers to the leftmost column of the design file tables or bill of materials. These identifiers are included for clarity and appear in square brackets. A complete assembly of the PowderBot machine is available as an IGES file in the Mendeley Data repository which can be opened by most CAD software packages. The complete assembly can be referred to during construction to check the location of every mechanical component.

### 5.1 Fabricating printed parts

All the 3D parts in the design were made and designed to be printed with a hobbyist-level single-extruder Fused Deposition Modeling (FDM) based printer. As such, no part requires any support material (non-soluble or soluble). All the printed parts in the following build instructions were sliced using Ultimaker Cura 4.13.1 (with material-specific default settings) and fabricated using the Ender3 V2. Most parts were printed with PLA material [A27], but the following parts were printed with semi-flexible TPU [A28]: D26 and D27.

### 5.2 Components as subassemblies

The next explanation is based on the separated subsystems of the PowderBot machine. Each subassembly is called “SA# XXX” where the number is the order defined and then its name (i.e., SA1.1 Pillar). For a fully step-by-step process to reproduce the building of PowderBot you can check the Build Instructions document which is plenty of images to follow the procedure easily.

**Fig. 10:**
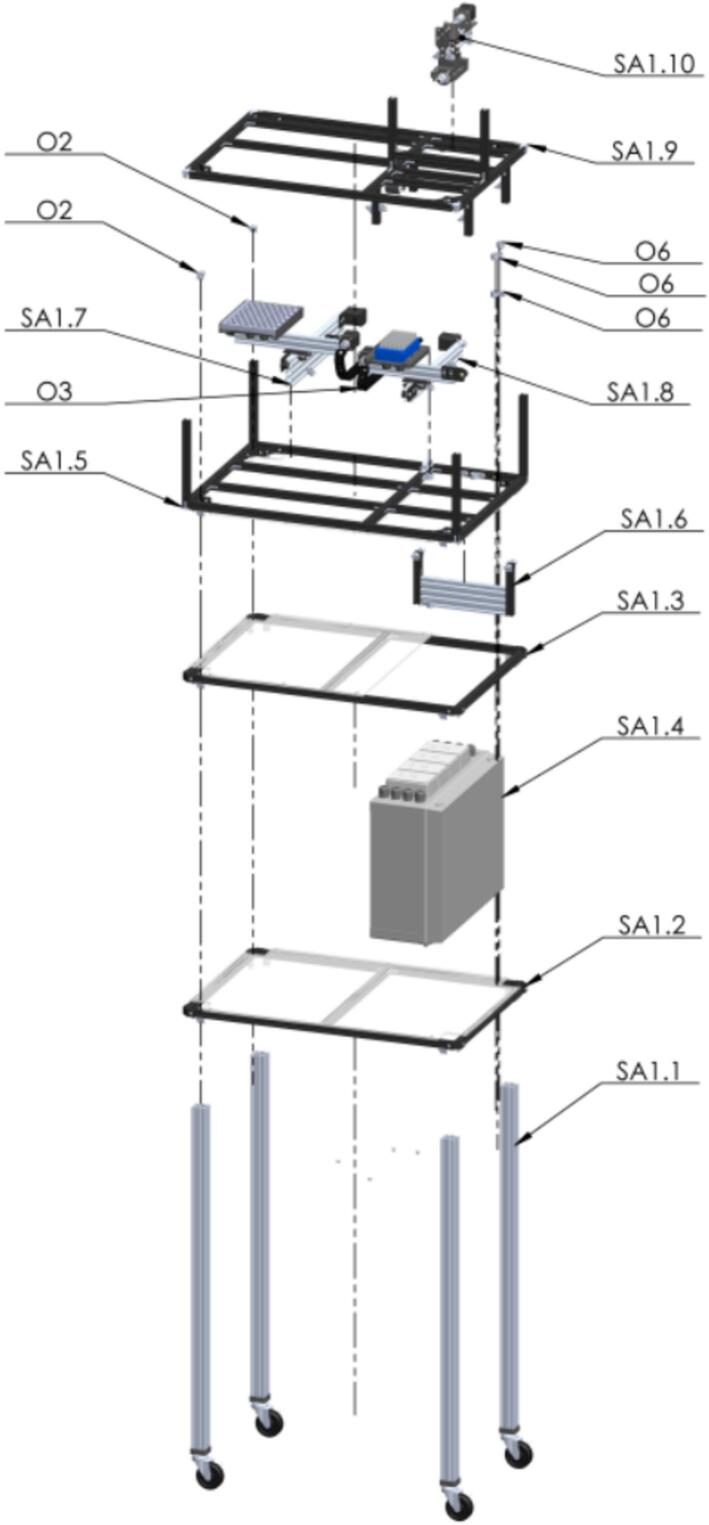
Exploded view of SA1 Frame CAD-based 3D model.

The first assembly to be made is the frame of the PowderBot machine Fig. 10. It is composed by several subasemblies such as Pillars, supports at different levels of altitude, X-Y axis for seed, drill, and plates movement, and the box for electronics.

**Fig. 11:**
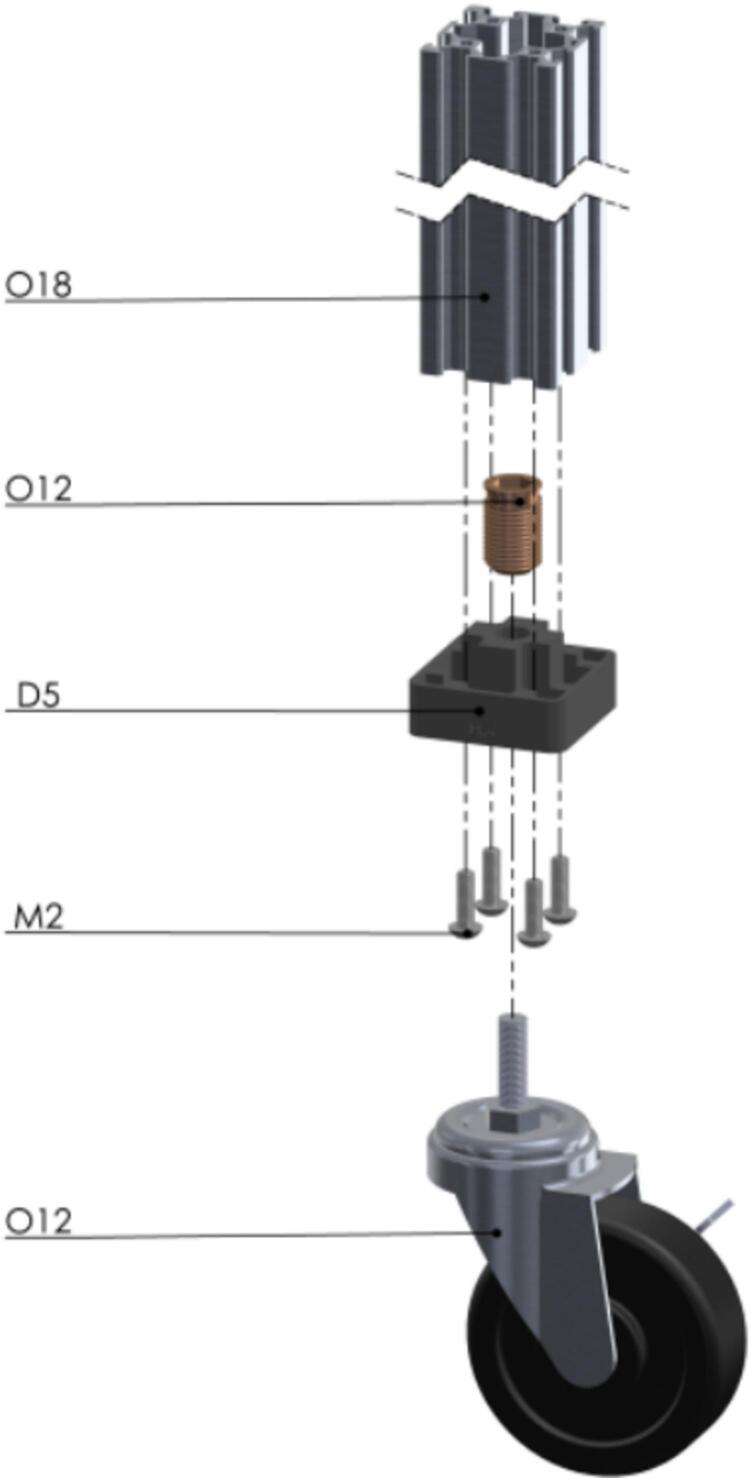
Exploded view of SA1.1 Pillar CAD-based 3D model.

So, let us start building the “legs”, called Pillars, to support the entire PowderBot structure Fig. 11.

**Fig. 12:**
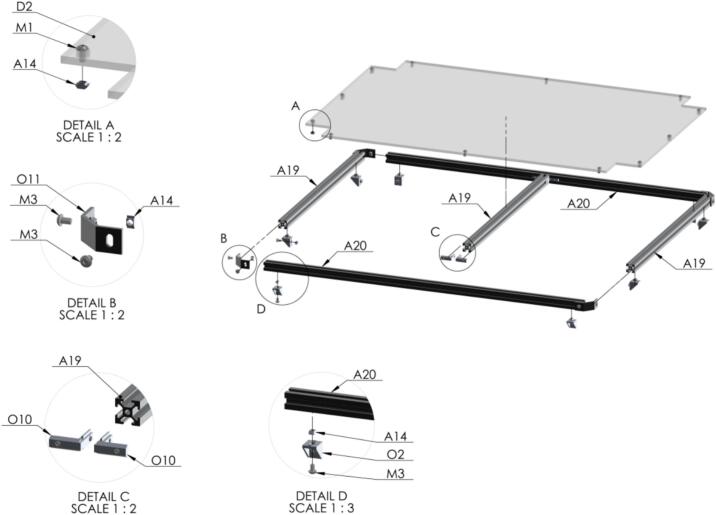
Exploded view of SA1.2 Support 1 CAD-based 3D model.

Here, the assembly for each level of the structure to support different equipment is going to be made. Three of them includes an acrylic piece which are attached in the laser cut designs. First, the support of the compressor at the lowest level Fig. 12.

**Fig. 13:**
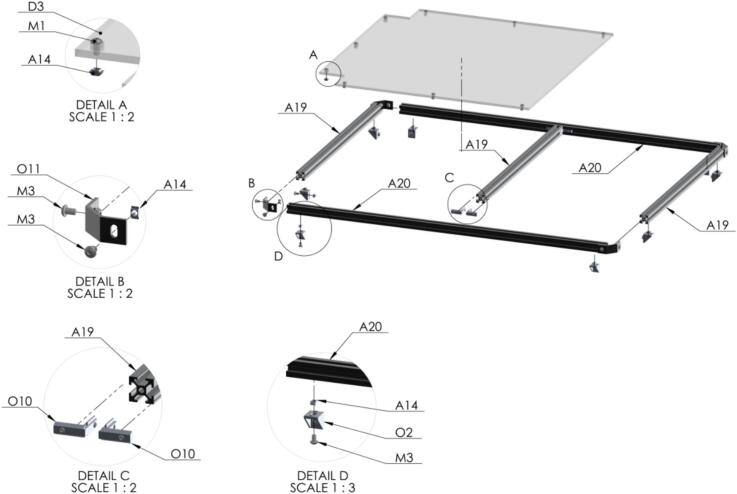
Exploded view of SA1.3 Support 2 CAD-based 3D model.

Similar than the previous step, the next level is built to support the vacuum cleaner machine Fig. 13.

**Fig. 14:**
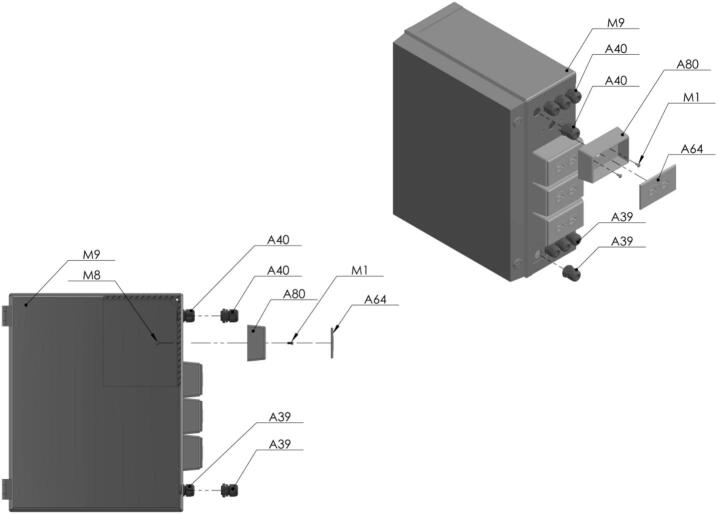
Exploded view of SA1.4 Control Box CAD-based 3D model.

Now, after following the Circuit Diagram for placing the circuitry and electronics for the control of the PowderBot machine, the box is ready to be accommodated between the first two levels built previously Fig. 14.

**Fig. 15:**
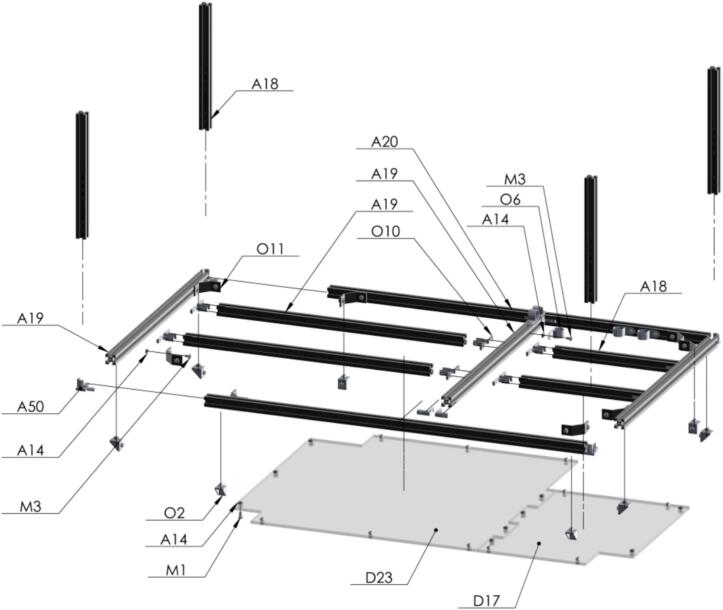
Exploded view of SA1.5 Support 3 CAD-based 3D model.

Next step, build the level to hold both the Seed and Sample Plates which will be mounted on two sliding aluminum v-slot linear rails Fig. 15.

**Fig. 16:**
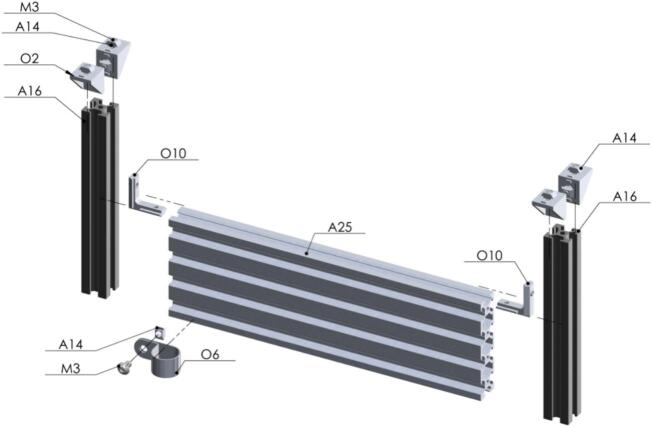
Exploded view of SA1.6 Support 4 CAD-based 3D model.

The triple set of pneumatic solenoid valve are mounted in the next support built on an aluminum profile rail Fig. 16.

**Fig. 17:**
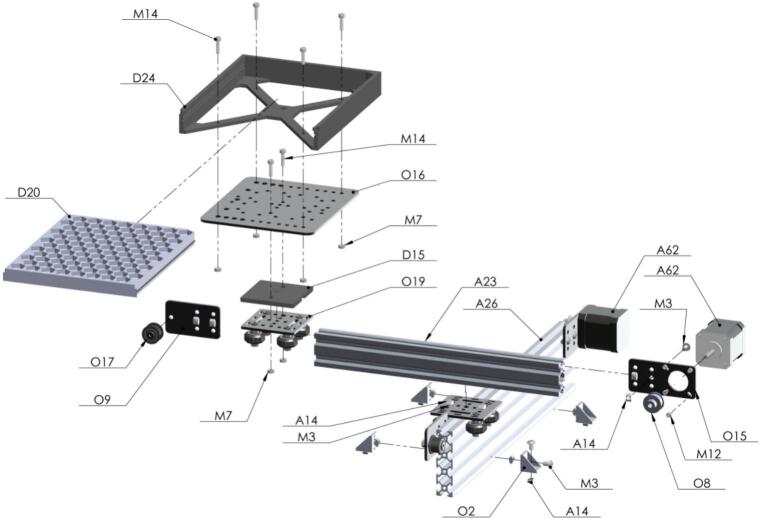
Exploded view of SA1.7 Seed Plate CAD-based 3D model.

Seed Plate is built using stepper motors for the XY movement. Some of the 3D printed parts are used here. Tight firmly but not too much to avoid cracking the plastic pieces Fig. 17.

**Fig. 18:**
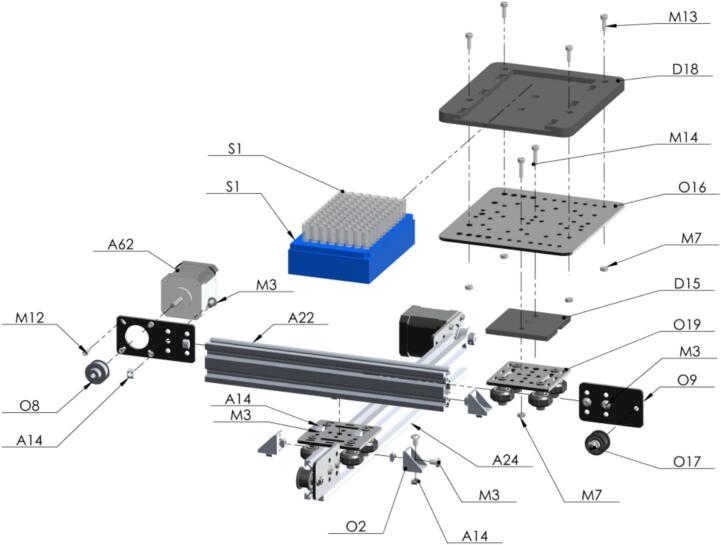
Exploded view of SA1.8 Powder Plate CAD-based 3D model.

Similar procedure as Seed Plate is going to be made with Powder Plate assembly Fig. 18.

**Fig. 19:**
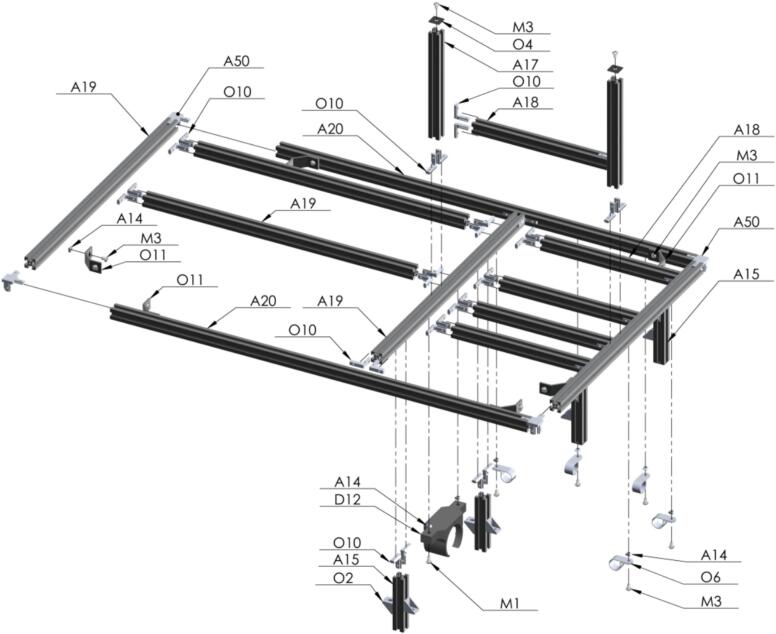
Exploded view of SA1.9 Support 5 CAD-based 3D model.

The last and upper level is going to be built to support the solenoid valves, PVC pipes, touchscreen display, light tower, and the rest of the stuff Fig. 19.

**Fig. 20:**
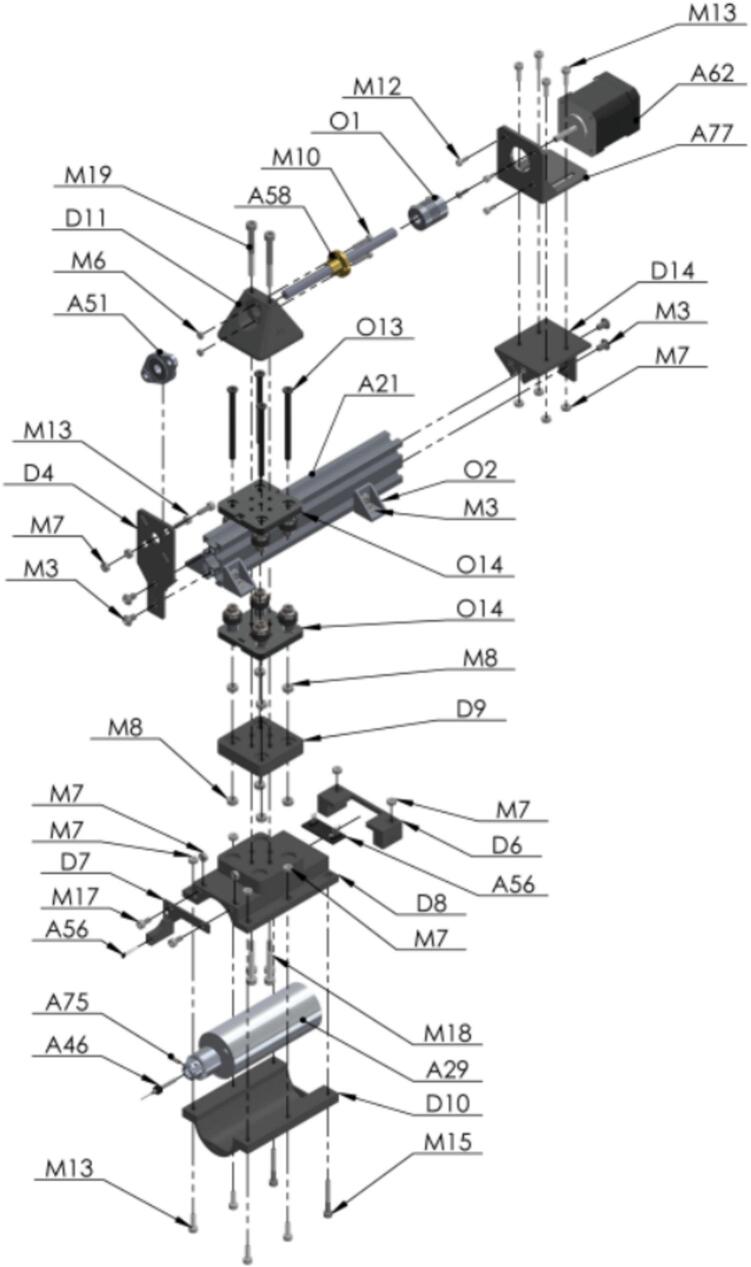
Exploded view of SA1.10 Custom Mini V Gantry Kit CAD-based 3D model.

The next steps are crucial because here is where the magic occurs at the moment when the seed is perforated by the bit drill [A46] to extract the sample in form of powder Fig. 20.

**Fig. 21:**
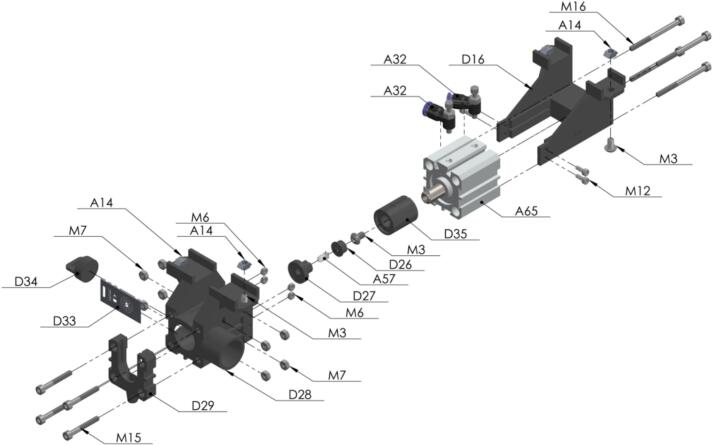
Exploded view of SA2 Seed Sampling Capsule CAD-based 3D model.

**Fig. 22:**
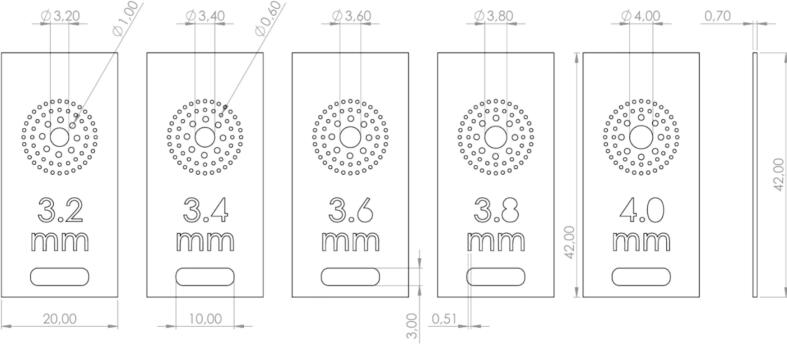
Front and lateral view of Seed Sampling Plate CAD-based 2D model.

Inside this capsule the seed is going to be grabbed and ready to be drilled. The air flow of the pneumatic cylinder [A65] must be adjusted to prevent damage on the seeds Fig. 21. Also, the metallic piece [D33] is selected according to the size of the seeds (a laser cut design is included) Fig. 22.

**Fig. 23:**
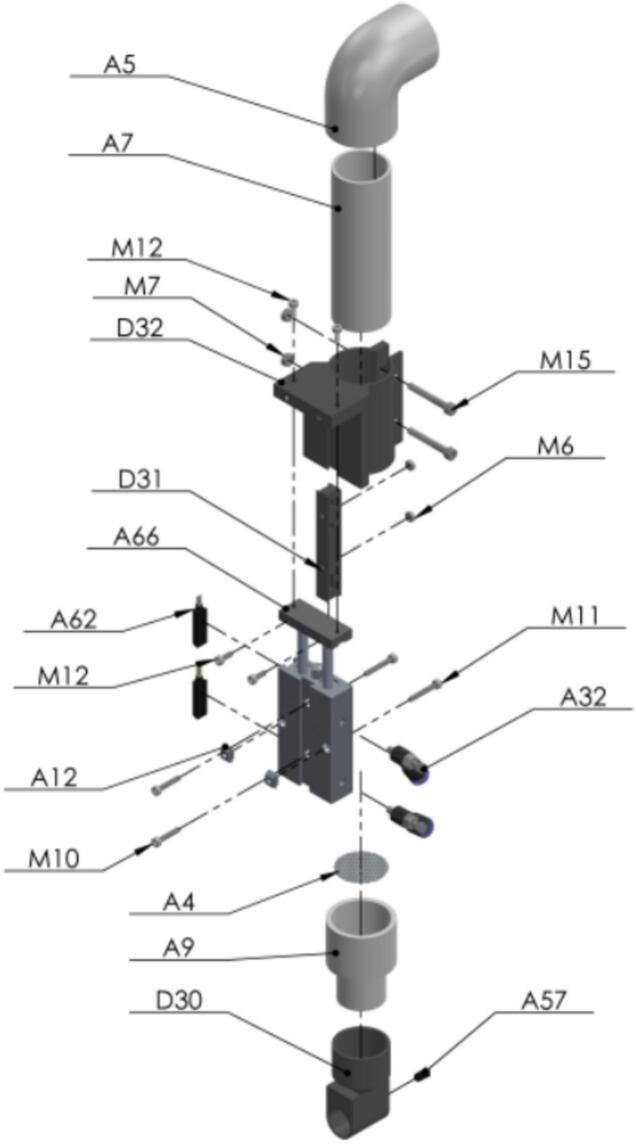
Exploded view of SA3 Custom Vacuum Funnel CAD-based 3D model.

Using this vacuum funnel the seed can be transported from the Seed plate to the Seed Sampling Capsule Fig. 23.

**Fig. 24:**
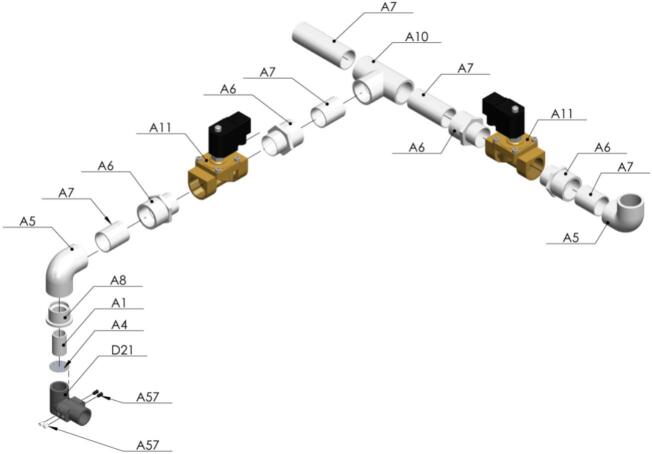
Exploded view of SA4 Vacuum System CAD-based 3D model.

Now, it is time to connect the vacuum system. No glue is needed to fix the PVC pipes nor the solenoid valves Fig. 24.

**Fig. 25:**
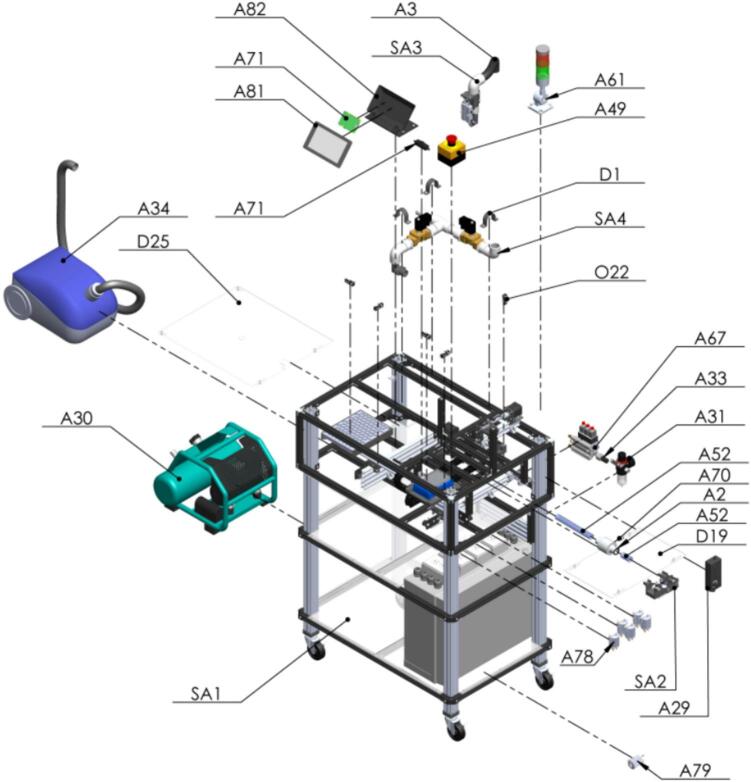
Fully exploded view of PowderBot CAD-based 3D model.

Finally, a complete assembly of the machine can be achieved after following the previous steps. Place the equipment accordingly and connect the outlet to the power supply Fig. 25.

## 6 Operation instructions

Here is a list of the flow cycle implemented in the PowderBot machine to explain how it works (for more information, see Operation Instructions document):

• Manual plates (seed and sample) filling.

• Manual plates positioning.

• Automatic repeated cycle as many seeds you filled (up to 96):

– Automatic sample tubes’ positioning.

– Automatic seed transportation.

– Automatic seed grab.

– Automatic powder extraction.

– Powder falls into correct tube.

– Automatic funnel cleaning.

– Automatic seed positioning after powder extraction

### 6.1 Manual seed/powder plates filling and positioning

**Fig. 26:**
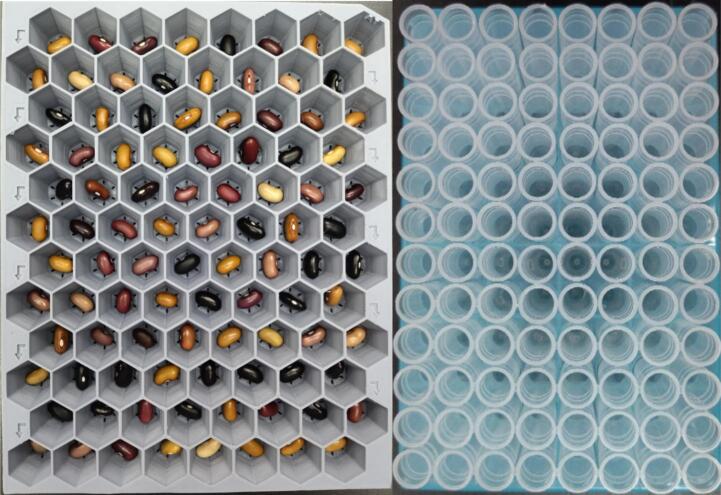
Seed Plate filled with 96 seeds on the left and Sample Plate filled with 96 tubes on the right.

Fill the Seed Plate with the seeds you want to extract and repeat the same process with the sample tube plate. Remember you have up to 96 seeds to be extracted in one process at a time Fig. 26.

**Fig. 27:**
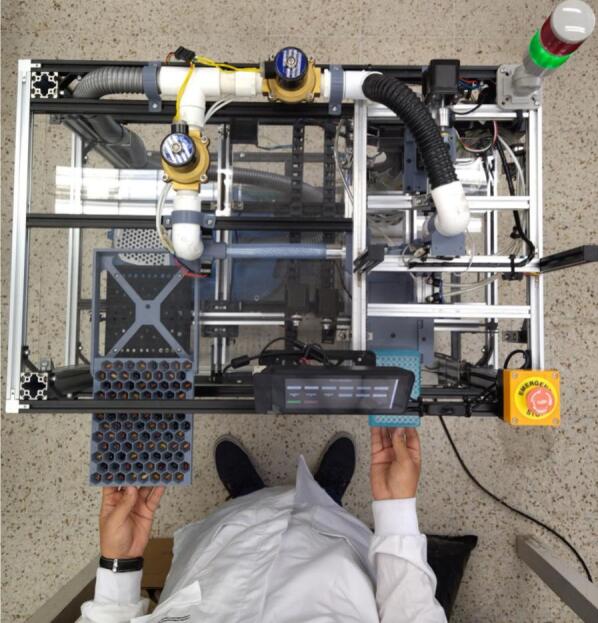
Manual Seed and Sample Plates positioning.

Insert the Seed Plate into the Seed Plate Support located in the left side until the stop barrier. Repeat the same process with the sample tube plate into the Powder Plate Support located in the right side Fig. 27.

**Fig. 28:**
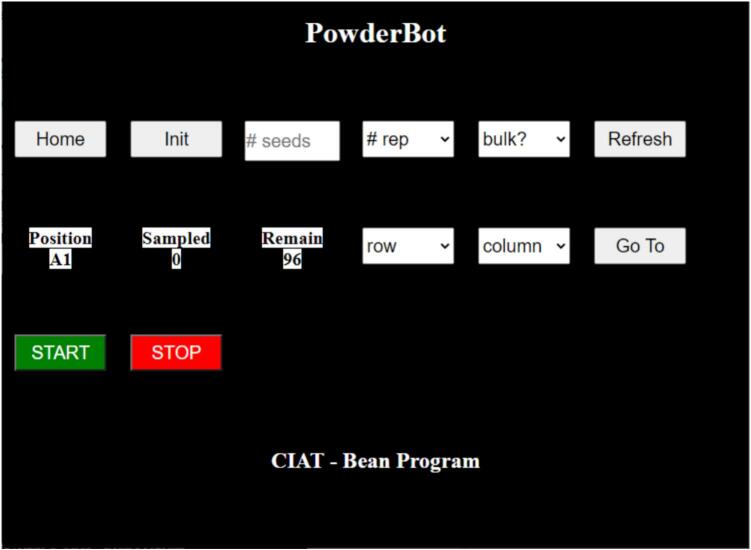
Web User Interface to operate PowderBot.

Once the machine is plugged in to the electricity and turned on, after a while the Touch Screen displays the web page with the options to be set Fig. 28.

### 6.2 Web app user interaction

The next list, representing every option available for the user to interact with the PowderBot machine:

• Home: Is a button with the option to move the axis to”zero” or”home” position for manual Seed Plate and Powder Plate positioning.
• Init: Is a button to move to another position as mentioned above but the difference is this represents the”A1″ or initial position to start the sample extraction process.
• #seeds: This is an input cell to introduce the number of seeds you want to extract. The range is from 1 to 96.
• #rep: This is a dropdown button to select one of the offered options. X1, X2, X3 represents the number of times you want to extract a sample from the same seed.
• Bulk?: This is another dropdown button to select between two options YES or NO. This is for the case you want to extract two or three seed samples into the same tube until it moves to the next sample tube.
• Position: As its name implies, this shows the current position of the plates in the format “row,column”.
• Sampled: Is a variable that changes based on the position of the plates. It is just for showing the number of seeds has been processed.
• Remain: Represents the number of seeds that are remaining to be processed.
• Row: Is a dropdown button to select any row you want to move the plates. The options are A, B, C, D, E, F, G, and H.
• Column: Is a dropdown button to select any column you want to move the plates. The options are 1, 2, 3, 4, 5, 6, 7, 8, 9, 10, 11 and 12.
• Go To: After”row” and”column” are set, you can press this button to move the plates to the desire position.
• START: As its name says, it is a button to be pressed and then start the extraction process. However, the process will be initiated if the next conditions are satisfied:
- The plates are in Init”A1″ position.
- The number of seeds”# seeds” is set between 1 and 96.
- The number of replications”# rep” is set between 1x, 2x or 3x.
- The bulk option is set to YES or NO.
• STOP: Is the button to stop the process but not immediately, if the machine is processing a seed, it will stop until finish the process.

### 6.3 Sample extraction process

**Fig. 29:**
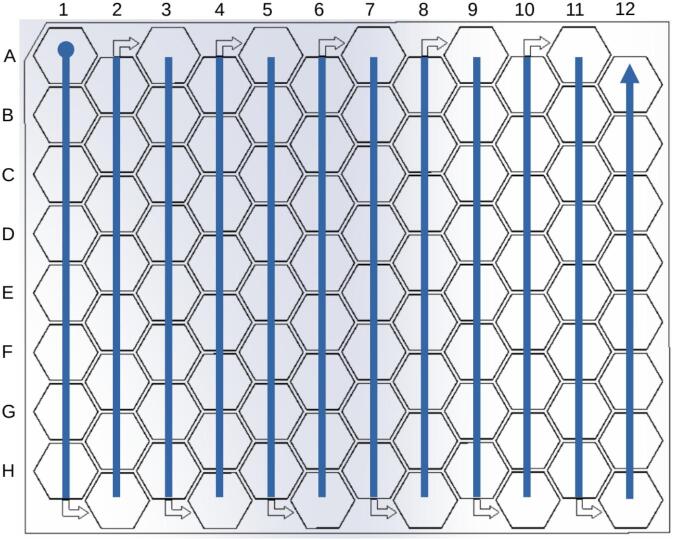
Diagram of Seed Plate in the sample extraction process. Serpentine sequence which “A1″ corresponds to the initial position while ”A12″ is at the end.

The PowderBot machine is initially positioned at Home position. After putting Seed Plate and Powder Plate in its respective positions then is also needed to follow the next steps in order to extract 96 seed samples (Fig. 29):

1. Press “Init” button.
2. Using the On-Screen keyboard put 1 to 96 in “# seeds” input text.
3. Click on dropdwon “# rep” button to select how many times you want to drill the seed. 1 to 3 times is available.
4. Click on dropdown “Bulk” button to extract two or three seed samples in one tube until it moves to the next sample tube. Otherwise, select NO.
5. Click on “START” button.

## 7 Validation and characterization

**Fig. 30:**
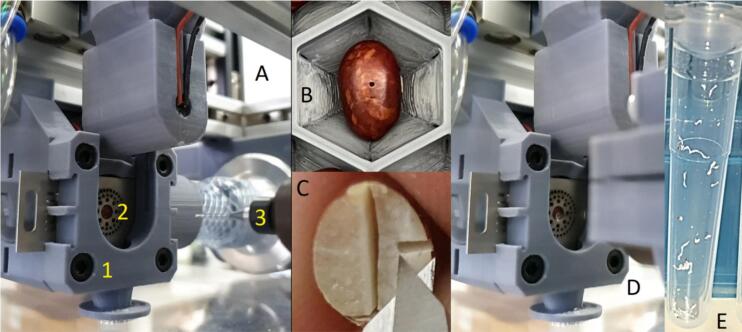
Seed powder extraction process, (A1) seed sampling capsule, (A2) seed ready to be perforated, (A3) 0.7 mm drill bit, (B) bean seed after hole drilling, (C) drill-hole depth (1.96 mm) on a bean seed, (D) seed drilled, (E) powder sample extracted.

To validate the use of the PowderBot, we performed bean seed powder extraction followed by molecular marker analysis and germination tests in seeds corresponding to 46 different common bean genotypes selected from a set from the Andean breeding line nursery (Vivero Equipo Frijol, VEF abbreviation, report VEF) [8] harvested in 2021. The size of the seeds are classified as medium / large (25–40 g / >40 g per 100 seeds) in the range of 12.8 to 18.3 mm of length, 7.4 to 10.1 mm of width, and their thickness usually falls between 4.6 and 6.9 mm. Two replicates of the extraction were evaluated. Furthermore, for each sample three powder subsamples were extracted, carrying out 1, 2, or 3 perforations on each seed (Fig. 30).

### 7.1 Validation of the methodology for obtaining seed powder with the PowderBot in the genetics laboratory

The PowderBot is an essential tool for the genetics laboratory of the bean breeding program at the International Center for Tropical Agriculture (CIAT), located in Palmira, Valle del Cauca, Colombia. It is used to extract DNA directly from seed powder. This DNA is essential for genotyping common bean lines selected for their agronomic interest or other traits relevant to the program. This technique is specifically implemented through marker-assisted selection (MAS).

#### 7.1.1 Seed DNA extraction for M.A.S

The Powderbot provided about 5, 10 and 20 mg of seed powder for 1,2, and 3 perforations respectively in each well of a 96-well sample plate (Assay Plate, 96 well, Costar ref.3795). Total DNA was extracted using the Alkaline extraction protocol, a fast and cheap methodology proposed by Klimyuk et al. (1993) [9] that allows rapidly handling a large number of samples (Garzon et al. 2007) [10]. This protocol uses two buffers, Buffer A (50 mM NaOH, 1 % Tween® 20) to degrade cell wall and Buffer B (100 mM Tris HCl, 2 mM EDTA, pH = 7.3) to neutralize DNA and bind proteins. About 100 µl of Buffer A were added to each seed powder sample. Samples were incubated in a water bath at 95˚C for 10 min. About 70 µl of Buffer B was added and samples were placed on ice. Subsequently, samples were diluted 1:20 (DNA (5 µl): H_2_O (95 µl)) and placed in a 96-well plate with a final volume of 100 µl. 5 µl of DNA template dilution was used for polymerase chain reaction (PCR) amplifications.

#### 7.1.2 Molecular marker analysis

To evaluate the utility of the DNA extraction method from the seed powder, the molecular marker for bc-3 was used. This marker recognizes a single nucleotide polymorphism (SNP) on chromosome 6 of common bean, related to bean common mosaic virus (BCMV) resistance. It has the same information content as the bc-3a marker [11], with the difference that bc-3 is a T_m_ (Melting temperature) change marker [12], so 3 primers are used in the PCR. Depending on the resistant (+) or susceptible (−) genotype, the marker will produce PCR amplification products of slightly different sizes. These can be distinguished by melting point analysis, which determines at what temperature the double-stranded DNA of the amplification product separates into two.

This marker has been widely used by the bean genetics laboratory for MAS to select individuals resistant to this virus in the CIAT bean breeding program crosses.

#### 7.1.3 Evaluation by PCR in real time with the molecular marker bc-3

The final volume of the PCR reaction is 15 µl using 5 µl of DNA dilution, 1.5 µl 1X PCR buffer ([10 mM of Tris–HCl (pH 7.2), 50 mM of KCl), 10 µM of each primer, 25 µM of each dNTP, 2.5 mM MgCl2, 1-unit *Taq* polymerase and completed the final volume with H_2_O MilliQ. PCR amplification was performed on a 384 Eppendorf Mastercycler *Pro* thermocycler, running the following program: an initial denaturation step at 94 °C for 3 min, then 40 cycles of denaturation at 92 °C for 15 s, annealing for 15 s (the temperature was specific to primer), and extension at 72 °C for 15 s, and final extension at 72 °C for 10 min. Melting point analysis for allele determination of the template DNA was performed with a fluorescence-detecting thermocycler (BioRad CFX384TM Real Time PCR System) with EvaGreen fluorescent dye (Biotium®, Fremont, CA). Fluorescent detection was carried out over 1 min at 95 °C and the melting curve step ramping from 75 to 95 °C in increments of 0.5 °C every 20 s.

To evaluate the quality of the genotyping results the Ct value (threshold cycle) (°C) produced of each sample was observed in comparison to the internal controls included in the PCR for verification.

#### 7.1.4 Cross contamination test

Two contrasting lines were selected (with the gene and without the gene for BCMV resistance). 92 seeds were used, distributed in two groups of 46 (attached as”Fig.ResultGenotyping92″ in Figures&Tables folder) likewise, 1, 2 and 3 perforations in each seed.

**Fig. 31:**
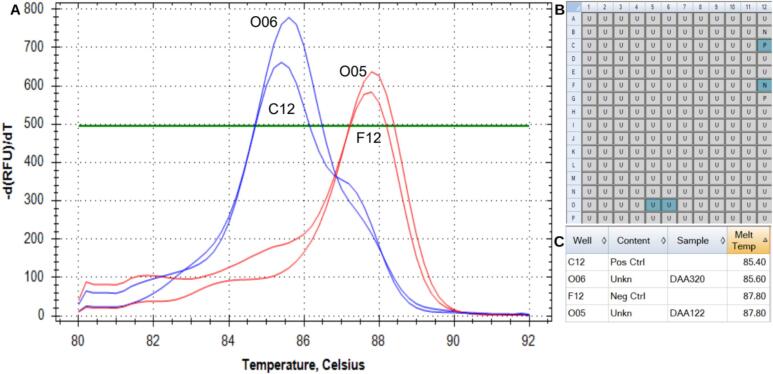
(A) Amplification of the contrasting samples (RFU stands for Relative Fluorescence Units), blue line R = resistant and red line S = susceptible, evaluation of cross-contamination, (B) well labels, (C) list of samples with their respective CT. Those melting peaks of PCR curves were generated by Bio-Rad CFX Maestro software (release 2.3 (5.3.022.1030)). (For interpretation of the references to colour in this figure legend, the reader is referred to the web version of this article.)

When analyzing the genotyping carried out on the two replicates, we observed that they were consistent, of the 46 lines, 1 is positive for the marker, indicating that the method of extracting DNA from seed powder works reliably. The melting curves obtained in the real-time PCR for each assay look clean and show only a single high curve (Fig. 31), indicating that there is no cross-contamination among the samples. Additionally, the extraction of seed powder from 1, 2 and 3 perforations does not change the result, which suggests that the least amount extracted per sowing cycle is sufficient.

**Fig. 32:**
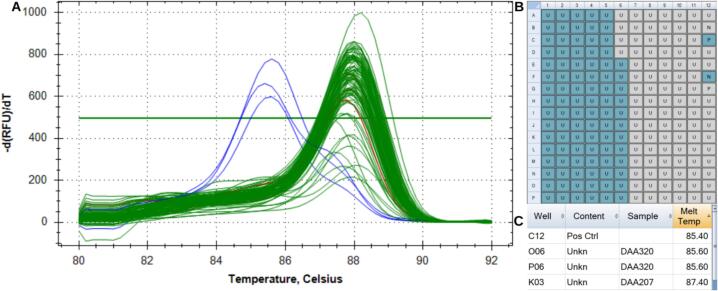
(A) Amplifications of the melting curves of the two replicas of the 46 lines VEF, (B) well labels, (C) list of samples with their respective CT.

Finally, the analysis of the real-time PCR melting curves obtained in each assay allows us to conclude that the performance of the method is exceptionally high, with an estimation of 99.9 %, in addition to being reliable and consistent (Fig. 32).

### 7.2 Analysis of bean seed germination levels after passage through the PowderBot

Seeds from Elite genotypes derived from the bean breeding program were run through the PowderBot to obtain seed powder samples for DNA extraction and subsequent molecular analysis for predictions on the trait of interest.

Under the suggested scheme of applying the PowderBot for a breeding program, we could rapidly and accurately provide key information to the breeder to make the decision to sow only pre-selected seed in nursery/field plots. This would save time and human and financial resources and accelerate genetic gains in beans or other given crops.

A basic requirement in this suggested process is that seeds perforated with the PowderBot maintain their germination capacity and still can develop into a normal plant.

After perforating the seed, they were delivered to the Tissue Culture and Cryopreservation Laboratory to quantify the percentage of germination. For each seed, the number and positions of perforations were noted.

**Fig. 33:**
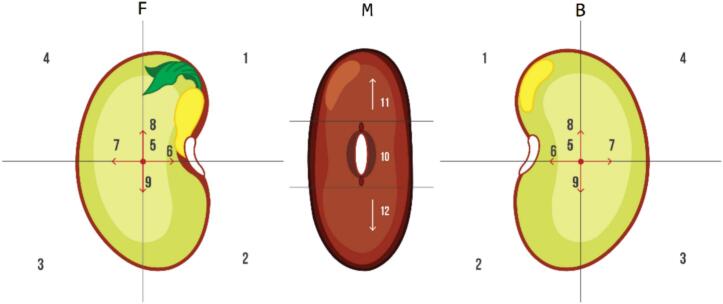
Scheme used to identify the position (F = Front, M = Middle, B = Back) and quadrant of the perforation(s) in the bean seed passed through the PowderBot. Twelve possible positions of the perforations in the treated seeds were observed.

Positions of perforations were recorded as F = Front, M = Middle, and B = Back, and the number quadrant under the field of view of a Nikon SMZ-1 stereomicroscope. The front of the seed is considered as the position in which the micropyle points to the right side (Fig. 33).

The seeds were then placed in trays of 50 cells, with washed and sterilized sand as substrate for germination. The seed trays with perforations at the bottom were placed outdoor on metal tables with a large eye mesh at the base to facilitate drainage, at greenhouse temperature (25 °C), with two irrigations per day. Three readings were taken at 8, 15 and 30 days after sowing. The first reading obtained at 8 days is considered as vigor or emergence capacity (germination initiation) and the following two evaluations as the formation of a complete viable plant (% effective germination). These two parameters (vigor and % germination) are fundamental components of the physiological quality of the seed [13].

#### 7.2.1 Treatment and preparation for seed germination assessment

Treatments with potassium nitrate (KNO_3_) and gibberellic acid (GA_3_) solutions are conventionally used in various species to accelerate and synchronize germination, however, the effect depends on the genotype [14]. Initially, tests were conducted to evaluate the effect of treating drilled bean seeds with the PowderBot for 24 h with aqueous solutions of 1 % KNO_3_ and 400 ppm GA_3_ and to determine if they helped accelerate the germination process. The bean seeds drilled and treated with these solutions were placed in a substrate of 1 part soil: 2 parts sand: 1 part peat moss for germination. A high percentage (96 %) of loss by rotting of the treated seeds was observed (data not shown). Due to this and based on the experience of the team, the condition was adjusted and once the seed was received from the PowderBot, the position and quadrant data of the perforation/treatment were read and obtained and immediately taken to trays with washed and sterilized coarse river sand. Moisture retention due to the presence of the soil and peat fraction in the substrate is probably the cause of seed rot loss. The use of coarse, washed and sterilized sand and the control of irrigation (twice a day) make the substrate in the trays drain and only maintain the amount of water/humidity necessary to activate the physiological processes that lead to the successful germination of the treated seeds. The percentage of loss due to rotting in the sand bed was minimal.

#### 7.2.2 Seed perforations

When analyzing the quadrant and position of the perforations (Table 3), regardless of the number of times they were made (1, 2 or 3), there was a higher percentage of occurrence at the front, 64.1 % with 1 perforation, 78.2 % with combinations of two perforations (from highest to lowest frequency 1F/1B, 2F, 1F/1M), and 83.7 % with 3 perforations (from highest to lowest frequency 2F/1B, 1F/2B, 3F).

**Table 3:** Frequency of location for seed perforations.

**Perforations**	**Front**	**Middle**	**Back**	**%**	**Seeds**
One (1)	59			**64.1**	59
		1		1.1	1
			32	34.8	32
Two (2)	1		1	**46.7**	43
	1	1		**4.3**	4
	2			**27.2**	25
			2	21.8	20
Three (3)	1		2	**33.7**	31
	2		1	**41.3**	38
	3			**8.7**	8
			3	9.8	9
	2	1		1	1
		1	2	3.3	3
	1	1	1	2.2	2

**Fig. 34:**
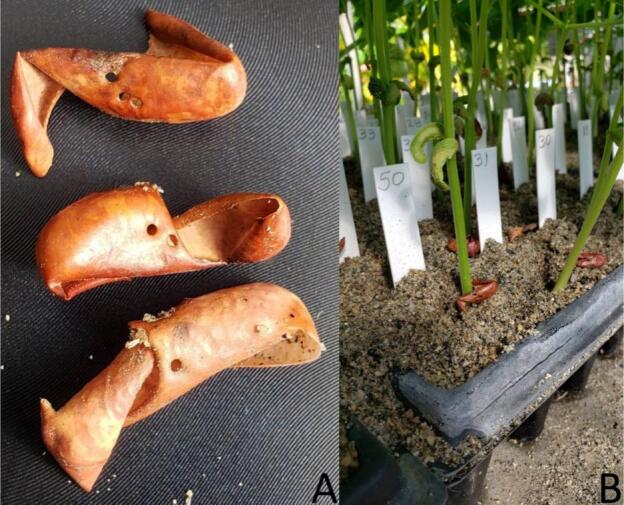
Seeds after 30 days of germination (A) seed coat perforated one, two, and three times in front, (B) trays with plants after seed germination.

During the adjustment of the template where the quadrant readings/data and position of the perforation(s) are recorded, some measurements of length x width x diameter of seeds of various sizes and shapes of beans were taken (data not shown). It could be observed that independent of bean shape and size, the highest frequency of perforations are in the front of the seed (Fig. 34).

The control seed, which are non-perforated seeds, and those with 2 perforations had a germination rate of ∼98 % (Table 4). Seeds with 1 or 3 perforations showed a germination rate of ∼90 % (Table 5). In this experiment, perforations in general lead to a slight reduction but does not show a dosage dependence on the germination and establishment rate.

**Table 4:** Comparison of the percentage of germination for bean seeds passed through the PowderBot between the control and 2 perforations evaluation.

	Control						2 Perforations					
	Rep1			Rep2			Rep1			Rep2		
	8	15	30	8	15	30	8	15	30	8	15	30
Germination	100 %			98 %			98 %			100 %

**Table 5:** Comparison of the percentage of germination for bean seeds passed through the PowderBot between 1 and 3 perforations evaluation.

	1 Perforation						3 Perforations					
	Rep1			Rep2			Rep1			Rep2		
	8	15	30	8	15	30	8	15	30	8	15	30
Germination	100 %			96 %	89 %	89 %	93 %	91 %	91 %	100 %		

Conventions for Table 4, Table 5:

• 8: First reading equals vigor at 8 days after sowing in trays with sand.
• 15 and 30: correspond to the percentage of germination at 15 and 30 days after sowing in trays with sand. If a seed has not developed at 30 days or has shown no response, it is considered dead or not viable.

**Fig. 35:**
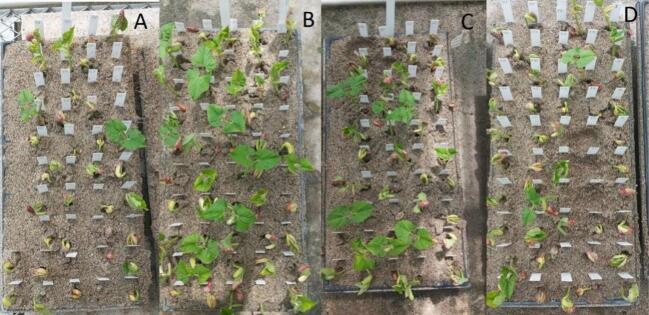
Evaluation of germination percentage at 15 days of VEF bean seeds (A) control without perforations; (B) passed through the PowderBot with 1 perforation, (C) with two perforations, (D) with 3 perforations.

Then, analyzing the response of the seeds with 1 perforation, seed rot was observed only in one genotype. The seed lot used in this test in general showed a high vigor and percentage of germination, which is a characteristic of a recently harvested lot (fresh seed) managed with seed quality standards. In all evaluations, independent of the number of perforations, at least one replicate (seed) showed high vigor and complete plant conformation (Fig. 35).

## 8 Conclusions

The application of the PowderBot method for a breeding program was validated. Genotyping results of seed-powder-extracted DNA show good quality, and no significant reduction in perforated-seed germination rates was observed.

The number of samples processed by PowderBot per cycle is 96 in around 30 min for 1 perforation per seed providing about 5 mg/sample. Also, the consistency of DNA samples across runs was validated as it was shown in the cross-contamination tests with clean melting curves obtained in the real-time PCR for each assay even with 1, 2 and 3 perforations in each seed.

Thus, the PowderBot is consolidated as a low-cost, assembled, and developed piece of equipment that supports genetic analysis on seed, with a facility to select and grow out these seeds. Breeders may identify seeds using the specific molecular marker(s) associated with the required trait(s). This generates benefits in breeding efficiency or quality control in non-commercial research programs. For a modest investment of around US$5,000, the genetic gains will be considerable.

To use the PowderBot, appropriate acknowledgment is mandatory. You may not use the material for commercial purposes. If you remix, transform, or build upon the material, you may not distribute the modified material.

Pilot tests with corn, rice, and wheat seeds were carried out with the PowderBot (data not shown), and in all cases we successfully obtained a sample (powder) for DNA extraction. Furthermore, it was possible to continue using the seeds until germination and obtaining complete and functional seedlings, all able to continue developing in the nursery and later in the field.

Uncited reference

[13,14].

## CRediT authorship contribution statement

**H. Díaz:** Writing – original draft, Validation, Supervision, Software, Methodology, Investigation, Conceptualization. **E. Macea:** Writing – original draft, Validation, Investigation. **R. Escobar:** Writing – original draft, Validation, Investigation. **S. Beebe:** Resources. **J. Tohme:** Funding acquisition. **B. Raatz:** Project administration, Conceptualization.

## Declaration of competing interest

The authors declare that they have no known competing financial interests or personal relationships that could have appeared to influence the work reported in this paper.
