# Supplementary figures and images for: PowderBot: An automated device for decision-making in crop breeding programs based on DNA extraction from seed powder
Source: HardwareX. 2025 Sep 22;24:e00706. doi: 10.1016/j.ohx.2025.e00706 (PMC12510228; doi:10.1016/j.ohx.2025.e00706)

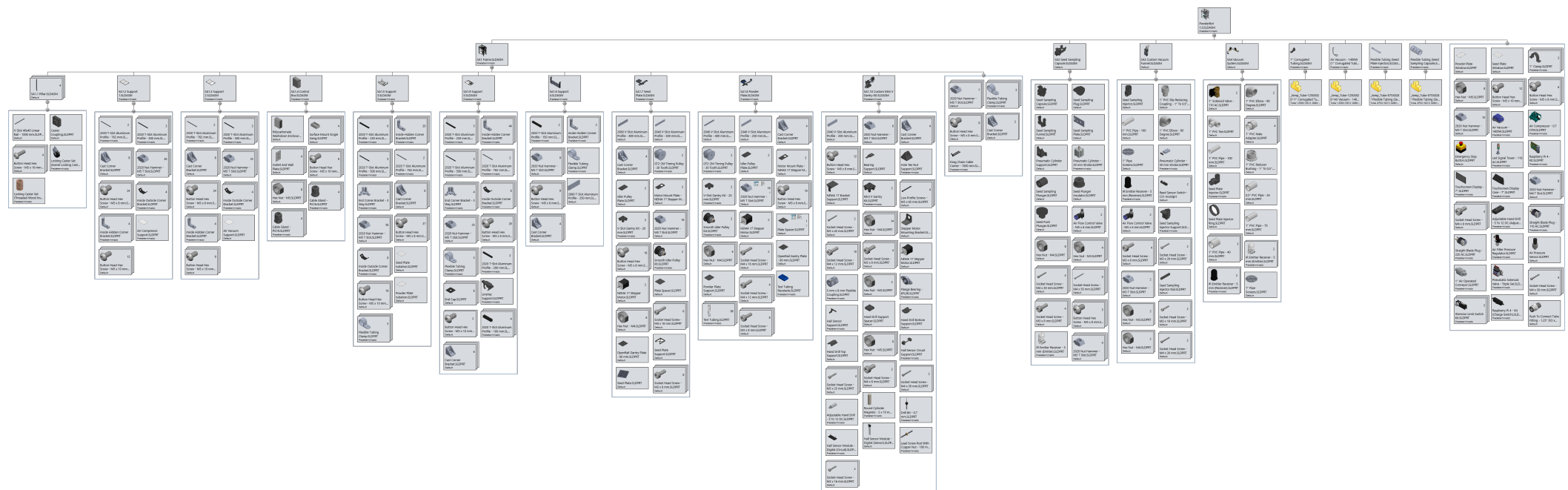

Supplement: Supplementary Data 1 [file mmc1.pdf]

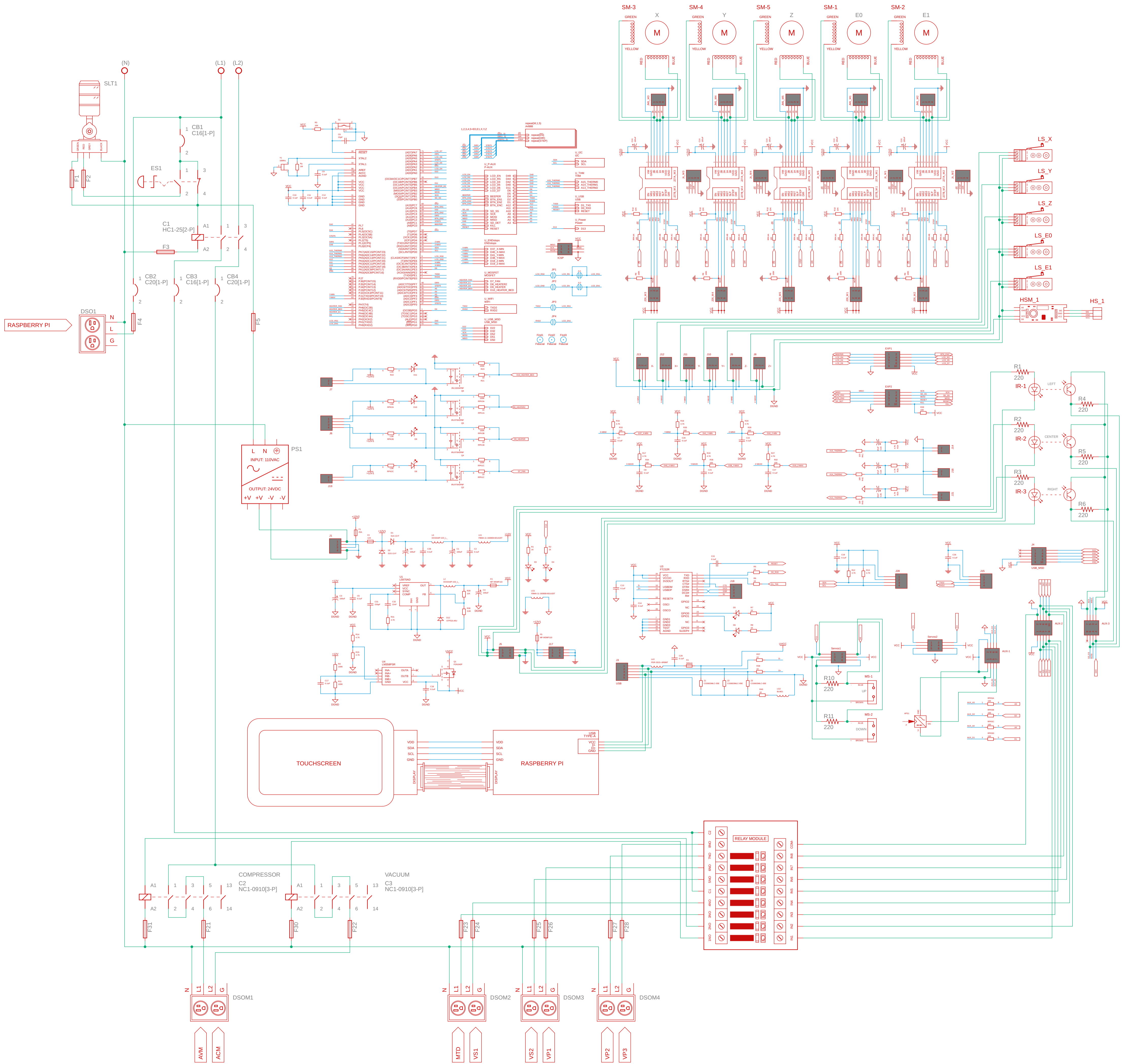

Supplement: Supplementary Data 2 [file mmc2.pdf]
